# Anatomy and Physiology

**Published:** 1837-07

**Authors:** 


### PART THIRD.

### Selections from the Foreign Journals.

---

### ANATOMY AND PHYSIOLOGY.

### *On Artificial Digestion.* By Prof. Müller and Dr. Schwann.

In the "Archiv für Anatomie und Physiologie" for 1836, we have two long and interesting papers on artificial digestion. The first is by Professor Müller and his assistant, Dr. Schwann, conjointly, and refers exclusively to the artificial digestion of coagulated albumen and boiled muscular fibre; the second is by Dr. Schwann alone, and has reference more particularly to the nature of the digestive process. The two papers occupy upwards of seventy pages; our limits permit us, therefore, to give but a very meager outline of some of the most important facts which they contain. Professor Müller was induced to undertake the investigation of artificial digestion by the publication, in 1834, of Eberle's "Physiology of Digestion," in which it is maintained that, although neither diluted acids nor mucus separately possess the power of dissolving organic substances, that property resides in acidified mucus, which is capable, not only of dissolving albumen and muscular fibre, but also of entirely changing their chemical nature, by converting them into osmazome and salivin. In the winter of 1834-35, Professor Müller had the satisfaction of convincing himself of the accuracy of Eberle's statements, and, in the winter following, he and Dr. Schwann undertook the following series of experiments:

They proceeded first to determine the action which diluted acids produce upon animal substances, and for this purpose small cubes of boiled muscular fibre and coagulated albumen, cut so as to present sharp edges and corners, were digested in common test tubes, with diluted acids, at a temperature of 30° R. At the end of twelve hours, digestion in dilute muriatic acid had produced no change in the appearance of the albumen or muscle, and, at the end of twenty-four hours, they had merely become somewhat more friable. Twelve hours' digestion in diluted acetic acid produced only a slight swelling of the cubes, and, at the end of twenty-four hours, the corners and edges still retained their sharpness. Longer digestion produced a gradual separation of the muscular fibres, but these were in no instance reduced into a pulpy mass. A like result was obtained from digestion with oxalic, tartaric, and lactic acids.

Drs. Müller and Schwann next proceeded to test the properties of acidified mucus. For this purpose, the mucous coat of the fourth stomach of the calf was carefully dissected off, and dried. The dried membrane was then cut into small pieces, and introduced into test tubes, half an inch in diameter, and so much distilled water added as to stand about three-fourths of an inch above the membrane, after the latter had ceased to absorb. Into two of these tubes from six to eight drops of muriatic acid were dropped, and into other two from twelve to fourteen drops of acetic acid. Another tube contained pieces of the mucous membrane, with water only; and another, the same quantity of water as the other glasses, with eight drops of muriatic acid, but no membrane. Into these glasses cubes of boiled muscle and coagulated albumen were introduced. After digesting for twelve hours at a temperature of 30° R., no particular change was found to have taken place in the two last-mentioned cases; but, where the acid had been added to the mucous membrane, the fragments of muscle had become greasy and puffy on the surface; they had lost the sharpness of their corners and edges, and their fibres could not be distinctly recognized. The albumen was puffy and translucent on its surface, soft and cheesy in the centre, and, by continued digestion, was completely softened and dissolved.

It appears to be immaterial whether pure mucus, or merely portions of the mucous membrane, be employed to prepare the "digestive fluid;" but Müller considers that the efficacy of the latter does not depend upon the membrane as such, but upon the mucus which is contained in its numerous follicles. The quantity of acid required to form a good digestive fluid is extremely small; about 3.3 grains being sufficient for two drachms of membrane and water taken together.

The question whether the solution of the coagulated albumen is a simple change of aggregation, or whether a chemical alteration ensues, next comes to be determined. To decide this point in as simple a manner as possible, sixty grains of dried mucous membrane were digested with acidified water for eighteen hours, at a temperature of 18° R.; eighteen grains of coagulated albumen, cut in cubes, were then introduced into the fluid, and allowed to digest for twenty-four hours, at a temperature of 20° R. By the digestion the albumen was deprived of its white colour, and became yellow, the edges and corners of the cubes were dissolved, and their consistency was so small that they broke down into pulp beneath the finger. The softened albumen was washed and kneaded in distilled water, and the emulsion thus formed thrown upon a filter. The same process was repeated twice with some slight alteration, but with the same result. The clear fluid which passed through the filter was tested in various ways, and the experimenters arrived at the conclusion that the albumen undergoes a chemical change, and is converted into osmazome, salivin, and a third peculiar principle, the nature of which it will require further experiments to elucidate.

It being therefore evident that acidified mucus produces a chemical change in the albumen, it became necessary to investigate the nature of the process. Some analogy seemed to exist between the process of fermentation and that of artificial digestion; and the question immediately presented itself whether, during the latter, there takes place any evolution of carbonic acid, or absorption of oxygen, and consequent oxygenation of the albumen. Experiment shewed that artificial digestion produced no change in the definite proportion of oxygen and nitrogen as they exist in the atmosphere. The process was unaccompanied either by evolution of carbonic acid or of any other gas, and proceeded quite as well in full and hermetically sealed tubes as in contact with the atmosphere. It is true that a very small quantity of carbonic acid was evolved during the process, but not more than would have been produced by any animal fluid. There was also a slight absorption of oxygen, which in all probability depended upon the tendency which osmazome has to acidify when in contact with the air. The fact, however, that digestion proceeds equally well when the contact of the air is prevented, and the very minute quantities of the evolved carbonic acid and absorbed oxygen, convinced our experimenters that the change produced in the albumen is not owing to oxygenation. They are still disposed, however, to consider the process as in some measure analogous to that of fermentation; and they regard the peculiar digestive principle which resides in the mucus as supplying the place of ferment. The nature of the process and the properties of the digestive principle are more fully considered in the second paper, of which we shall now give an abstract.

Mucus, it has been ascertained, is insoluble in acids: Dr. Schwann found, however, that filtration did not deprive the digestive fluid of its peculiar properties, and consequently the opinion that these were derived from the mucus, was considerably weakened. To elucidate this subject, about two pounds of the mucous membrane of the third and fourth stomach of the ox were cut into small pieces and mixed with water, to which about two ounces of muriatic acid were added, and the whole was then digested for twenty-four hours, at 32° R. The greater part of the membrane was dissolved, and there resulted a troubled opaquish liquid, mixed with undissolved mucus and shreds of membrane. After filtration, there remained about three-fourths of a quart of a dullish, yellow fluid, which, even after standing for months, deposited no sediment, and contained 2.75 per cent. solid contents.

This liquid we shall term A. The undissolved residue was treated with fresh water and acid, and gave about half a quart of filtered liquid, which we shall term B; and the proceeds of a third digestion we shall term C. The acid contents of each of these three solutions were nearly equal; two drachms of each requiring about 2.5 grains of carbonate of potash respectively for saturation. Cubes of coagulated albumen were introduced into glasses containing portions of each of these solutions. Twelve hours sufficed for the liquor C to finish the digestion; it proceeded slower in A; but the power was increased by the addition of acidified water, while a like addition diminished that of C; and hence it appears that the digesting principle was contained in greater quantity in A than in C.

The next question to be solved was, whether acid was necessary for carrying on the process of digestion, or whether it served merely to call into action some other principle, which, once formed, was sufficient to carry it on without further assistance. To ascertain this point, the digestive liquor was neutralized with carbonate of potash: the digestive power was thereby destroyed, but was again restored by the addition of acid. It has already been shewn that acid alone possesses no digestive power: on what principle, then, does the acid act? Does it, in the first place, serve merely as a menstruum of the digestive principle; or, 2dly, does it form with it a peculiar chemical combination, analogous to the acid salts, which then effects digestion; or, 3dly, is the acid necessary for the solution of the product of digestion; or, 4thly, is it decomposed during the process to enter into combination with the product; or, 5thly, does it, by simple contact, predispose the digestible bodies to decomposition, without undergoing decomposition itself? In order to solve the first question, rather more than the half of the acid of a portion of the liquor C was neutralized; but still leaving a decided acid reaction, and without producing opacity in the liquid. The digestive power was destroyed, and the question answered in the negative.

Were the second the true view of the case, it would be necessary to shew that the acid always bears a relative proportion to the quantity of digestive principle contained in any menstruum. The contrary was proved by the following experiment:—To two drachms of distilled water 4.8 grains of the liquor A were added, and a like quantity to two drachms of acidified water, the acid of which was in the same proportion as in the normal digestive liquor. The degree of dilution of the digestive principle was in both cases alike; the quantity of acid alone varied. After twenty-four hours' digestion, no change had been produced in the albumen by the first mixture; by the second, it was completely dissolved. The same experiment is considered to disprove the third proposition; and the fourth is disproved by the fact that the same quantity of carbonate of potash is required to saturate the liquor after as before digestion. We are therefore forced to accede to the opinion that the acid acts merely as the predisposing agent of the decomposition, exactly in the same way as the diluted acids act in the conversion of starch into sugar when boiled.

We have now to enquire in what manner the digestive principle itself acts in producing artificial digestion. There are only two ways: either it must be by one of the chemical processes by which bodies are dissolved, or else it must be referred to the power of simple contact, analogous to that exercised by yeast during the process of fermentation. To determine this point, the following experiments were instituted:—It was first necessary to ascertain the quantity of digestive principle required for the digestion of a given quantity of albumen; and, for this purpose, two drachms of the digestive liquor, A, were introduced undiluted into a tube, *a;* into another tube, *b*, were introduced two drachms of acidified water, containing eight per cent. of the same digestive liquor; a tube, *c*, contained four per cent.; *d*, one per cent.; *e*, one-half per cent.; *f*, one-fourth per cent. of the same liquor; and, lastly, *g* contained simple acidified water. Into each of these glasses pieces of coagulated albumen were introduced. After twelve hours' digestion, the albumen in the glasses *b* and *c* was entirely dissolved; in *a* and *d*, it was very soft and translucent, but the form of the pieces was still easily distinguishable. In *e*, and even in *f*, a very manifest change was observable in the albumen; but in *g* it remained unaltered.

Again, to ascertain in what relation the digestive power of diluted and of undiluted digestive liquor stands to each other, the following experiment was performed: —A drachm of moist coagulated albumen was rubbed up with 4.8 grains of the liquor A, and two drachms of acidified water were afterwards added; a like quantity of albumen was introduced into two drachms of the undiluted liquor; and both mixtures were then digested for twenty-four hours. The albumen in both cases had almost entirely been dissolved. Thus, 4.8 grains of digestive liquor, containing 0.11 grains of solid ingredients, had sufficed to cause the solution of sixty grains of moist albumen, or of about ten grains of solid matter: that is, one grain had sufficed to produce the decomposition of 100 grains; a proportion which is known to occur only in the process of fermentation. In a set of experiments instituted to prove that the digestive principle is itself decomposed by the process, it was clearly ascertained that the digestive power of the liquor was destroyed, or at least greatly weakened by it; but it is impossible, in the present state of our knowledge, to determine the intimate nature of this change.

It still remains to determine the nature of the digestive principle. Eberle identified it with mucus: if such were the case, a saturated solution of mucus should possess digestive powers in an eminent degree. To ascertain this point, Dr. Schwann digested pure mucus, obtained from saliva, with a large quantity of acidified water: the greater portion of the mucus remained undissolved. Into the solution thus obtained albumen was introduced; but, after several days' digestion, no change was perceptible. On the other hand, he found that, when mucus, obtained in the same manner, is digested with a small quantity of acidified water, a solution is obtained which possesses digestive properties; he therefore infers that the digestive principle is not identical with mucus, but is either a new principle formed by the action of the acid upon the mucus, or some other peculiar principle which exists in the latter in small quantities.

It was next necessary to determine the nature of this peculiar principle, and for this purpose its behaviour towards various reagents was tested in the following manner:—To a quantity of the liquor, B, some reagent capable of producing a precipitate was added, and afterwards a bit of coagulated albumen. If the digestive power was found to be destroyed, some other reagent capable of neutralizing the first was added; and, if the power were thus restored, it became necessary to determine whether it had been recovered from the precipitate or from what remained in solution. For this purpose, the precipitate was collected upon a filter, and it and the filtered solution tested separately, according to general chemical rules. The results of these experiments show the digestive principle to be possessed of the following properties:—It is soluble in water, in diluted muriatic and in acetic acids; it is decomposed by alcohol and the boiling temperature; it is completely precipitated by acetate of lead, both from its acid and neutral solutions, and from the latter by corrosive sublimate. It is not precipitated by ferro-cyanate of potash, and infusion of galls destroys the digestive power, probably by forming an insoluble precipitate. These properties mark the digestive principle as a peculiar substance, distinct from osmazome, mucus, albumen, salivin, or casein. Dr. Schwann has hitherto been unsuccessful in his endeavours to isolate it, but the following plan of analysis is that which he considers as most likely to lead to that result. Precipitate with ferro-cyanate of potash, and filter. The filtered liquor contains osmazome, salivin, and the digestive principle. Neutralize with carbonate of potash, and precipitate with corrosive sublimate. Osmazome and the digestive principle are precipitated; salivin remains in solution. Wash the precipitate with great care, then add water acidified with muriatic acid in the same proportion as it exists in the digestive liquor, and decomposed by sulphuretted hydrogen. The digestive principle, and perhaps also the osmazome, are redissolved; but the great difficulty would consist in separating them. One of the best tests for the detection of the digestive principle is the property it possesses of coagulating milk; although, till we succeed in isolating the principle, some doubt must always obtain as to its being the agent of coagulation. Two grains of the digested liquor, A, sufficed to produce the coagulation of two drachms of cow's milk, when heated in the vapour bath, in one minute and a half; one grain required two and a half minutes; and half a grain four minutes, to produce the same effect. As a temperature o. 80° R. was found sufficient to destroy the coagulating power, it is evident that it could not be ascribed to the small quantity of acid contained in the digestive liquor.

In conclusion, Dr. Schwann remarks, that these experiments are not to be considered as applicable to the digestion of every substance, but to such only as are digested in the same manner as coagulated albumen. To the latter, his experiments showed, belong the fibrine of the blood, the raw and boiled muscular fibre of the ox, the flesh of roast beef and roast veal; but the digestive liquor produced no other change upon gelatine, casein, starch, and gluten, than what simple acidified water equally produces. It is worthy of remark, however, that the products of the digestion of these substances (if we except starch,) with simple acidified water, agreed, in their more essential characters, with those ascribed by Tiedemann and Gmelin to those produced by their natural digestion in the stomach; and hence Dr. Schwann is inclined to believe that curd, gelatine, and gluten may be dissolved and digested simply by the free acid of the gastric juice. This explanation, however, will not suffice for the digestion of starch, which, according to Tiedemann and Gmelin, is converted by digestion into gum and sugar: no such change is produced by simple digestion with acidified water; but, if boiled starch be digested for twenty-four hours with acidified saliva, no change of colour will be produced by the addition of iodine in the filtered solution, which will be found to contain sugar and gum. This change agrees with that which, according to Tiedemann and Gmelin, is produced by natural digestion; and hence, if a corresponding change does not ensue from the action of the gastric juice, a solution of the problem will perhaps be found by taking into consideration the quantity of swallowed saliva.

*Müller's Archiv. Jahrgang,* 1836. Heft i.

---

*Observations on Weber's Experiment on the Power by which the Head of the Thigh Bone is retained in the Acetabulum.* By DR. LAUER, Assistant Surgeon at the General Hospital, Hamburg.

[A FULL account of Weber's experiments has been given in a preceding number of this Journal. (Vol. II. p. 236.) His proposition is, that the head of the thigh bone is retained in situ, not by the power of the muscles or ligaments, but by the pressure of the surrounding atmosphere, and he draws from this several deductions bearing on the physiology and pathology of the hip-joint. Dr. Lauer repeated the experiment under the direction of Dr. Fricke with nearly the same results, but thinks that the conclusions drawn from it by Weber are deficient in proof, and of much too general a nature.]

The force exerted by atmospheric pressure on the head of the thigh bone, is equivalent to the weight of a column of air the base of which is equal to the superficial area of the head of the bone, or to the weight of a column of mercury of the same base and of a height equal to that of the column of an ordinary barometer. Supposing now that the superficial area of that part of the head of the bone which is covered with cartilage is from five to seven inches square,* and fixing the average height of the column of a barometer at 28.409 Vienna inches, and taking the weight of a column of mercury of the given height and a quarter of an inch in diameter, at 12.6 pounds, we find the force with which the pressure of the atmosphere acts on the head of the bone, to be equal to from 63 to 88.2 pounds. Now admitting that the head of the thigh bone is retained in the acetabulum by no other means, it would require a power equal to this weight, and acting in the direction of the neck of the bone, to separate the head of the thigh bone from the acetabulum, which, plainly, is not the case.

How different is a relaxed dead muscle from a living contractile one! How different at least with respect to sensation! Leaving out of view the proper con-

* The superficies of the largest head examined by Dr. Lauer was 6½ square inches.

tractile power of the latter, its mere vital tone, a property which the former does not possess, must contribute to this object. When a temporary contraction of the muscles of the hip-joint, whether effected through the agency of the will or by other causes, produces a transient or permanent shortening of the limb, and, vice versâ, a relaxation of these muscles is followed by its elongation—facts first pointed out to me by Dr. Fricke, and of which I have daily opportunities of convincing myself—are we not justified in assuming, that muscles in the normal condition, even when at rest, are, by means of their vital tonicity alone, capable of at least contributing to retain the head of the thigh bone in the acetabulum? In making these observations I am far from denying the influence of atmospheric pressure in effecting the same object. Can the shortening of the limb which occurs in the first stage of coxarthrocace, before the head of the bone has undergone any change of form or situation, and which admits of being demonstrated by the measuring rule, be explained by a supposed partial increase of atmospheric pressure? Can the shortening of the lower extremity produced by contusion of the muscles of the hip-joint, and removed by the application of leeches or cupping glasses, be accounted for by variations in the pressure of the atmosphere? Are we to attribute to the same cause the elongation of the thigh observed in that form of disease to which Dr. Fricke has applied the term coxalgia? In the former cases, however, the muscles are found to be evidently tenser and harder to the touch, in the latter, laxer and softer.

Weber's experiment has proved that atmospheric pressure is in itself sufficient to prevent the sinking of the head of the bone out of the acetabulum, when the limb hangs at rest; but would it, even were its power much more considerable, prevent the head of the bone from slipping out in violent motions, as for instance, in forcible divarication of the lower extremities, which some men can perform to such a degree without consequent luxation, that the outstretched axes of the lower extremities fall in a straight line and form an angle open superiorly? Here the muscles must certainly cooperate in a very remarkable manner, sometimes one portion, sometimes another, acting more powerfully according to the necessities of the case.

It is well known that travellers on very lofty mountains suffer many and obvious inconveniences from the diminution of atmospheric pressure, but I do not recollect that relaxation of the coxo-femoral articulation has been classed among them. It is true, a sense of fatigue has been very frequently noticed, but this in general is speedily removed by a short repose, and can scarcely be attributed to any peculiar relaxation of the hip-joint. Yet Saussure's barometer did not stand higher than sixteen Paris inches on the summit of Mont Blanc, and on Chimborazo, at a height of 6004 meters, Boussingault's stood no higher than 13 inches 8½ lines.

As to the ligaments, they are, with reference to this connexion, of much less importance. Still, in our experiments, we found that after boring through the acetabulum, not more than two thirds of the head of the thigh bone descended from the socket, as long as the capsular ligament remained entire; but when this was divided, the ligamentum teres opposed no further obstacle to the descent of the head of the bone, only, however, when this was effected by the weight of the limb itself, or (when the body lay horizontally) by traction acting downwards and pretty much in the direction of the axis of the acetabulum. But, if we attempt to make the head of the bone slip out of its cavity by forcible abduction, as occurs in cases of luxation from violence, the ligamentum teres certainly offers some opposition, and a complete removal of the head of the bone from the socket is effected only by employing a force capable of rupturing the round ligament.

If we turn to the use which Weber has made of his discovery in explaining spontaneous dislocation, we learn from him in the next place, that, occasionally, in men otherwise healthy, the head of the bone suddenly sinks out of the acetabulum. As far as I can recollect, no observation of this kind has been hitherto made. He says further, "Since the head of the bone is not, as far as we have seen, retained in the acetabulum by the power of the ligaments, we should not, in attempting to explain the origin of this affection, assume that the ligaments must be elongated or generally changed, before the head of the bone can quit the acetabulum. We have seen that this has occurred when air has got into the cavity of the joint above the head of the bone. But it is not necessary that the substance which gets in should be air. It may be a fluid secreted into the joint by the blood-vessels or it may be a solid substance growing within it. In proportion to the increase of the fluid or other substance formed there, the head of the bone sinks by its own gravity, without being necessarily forced down, and without any resistance being offered on the part of the ligaments."

It is certainly true, that the head of the thigh bone may sink to a certain extent out of the acetabulum without any preceding change in the ligaments; but before this happens completely, and in such a manner as to give rise to actual spontaneous luxation, they must certainly have suffered some alteration. It likewise cannot be denied, that a fluid situated in the cavity of the acetabulum above the head of the bone, or a solid substance formed in the same situation, may give rise to a dislocation of the same kind; but abstractedly from that point, that this occurs only in the rarest cases of spontaneous luxation, the bone does not sink out of the acetabulum during this process by its own weight, but is actually forced out. It is true that a few drachms of fluid are not sufficient to overcome the force of atmospheric pressure; but a fluid of this description is incompressible, and where it is, the head of the bone cannot be, and even the greatest degree of force capable of being exercised by the muscles is insufficient to retain it in the cavity. Weber himself says the same thing in an indirect way, and therefore involves himself in a contradiction; for he says, "the sinking of the head of the bone is in proportion to the increase in the quantity of the fluid." This case is of course quite different with air which has forced its way through a hole in the acetabulum; for this is in connexion with the whole atmosphere, and therefore presses the head of the bone downwards with the same power as it is pressed upwards by the operation of the same medium, so that the limb can now obey the impulse of its own gravity.

The experiments in question, according to my opinion, afford the following anatomical, physiological, and pathological results:

1. The head of the thigh bone completely fills the acetabulum, and the opposed surfaces are adapted to each other.

2. The pressure of the surrounding atmosphere is to be classed among the means by which the lower extremity is kept in apposition with the trunk of the body.

3. A spontaneous sinking of the head of the thigh bone out of the acetabulum may occur under certain circumstances without any preceding change in the ligaments.

*Zeitschrift für die Gesammte Medicin.* Band. ii. Heft iii.

---

*A single Artery for the two Ventricles of the Heart, Cyanosis.*

M. BARON presented to the Academy the heart of an infant, born on the 22d of November. It was brought to the Infirmary on the following day, in a state of general cyanosis. The beatings of the heart sounded obscurely, and there was a *bruit de soufflet.* No other symptom was observed until the 2d of December, when diarrhœa and vomiting occurred: the child died on the 7th of December. On examination after death, the heart was found to possess but one artery, the aorta, which arose from both the ventricles. Between these two origins, the interventricular septum formed a kind of spur (*éperon?*) There was no opening in the right ventricle for the pulmonary artery. The arch of the artery gave off from its convexity the usual branches. From its concavity proceeded a branch, the ductus arteriosus, which divided into two branches, forming the pulmonary arteries. At the point of the bifurcation of these two branches arose another, which was the pulmonary artery; it passed obliquely towards the base of the right ventricle, where it ended in a blind cavity. An injection was forced into this vessel, but the liquid did not pass into the ventricle.

*Bulletin de l'Academie Royale de Médicine.* No. 5. 1837.

### *On the Action of the Acetate of Lead upon the Animal Economy.*
By Professor C. G. Mitscherlich, M.D.

After some preliminary observations, Dr. Mitscherlich proceeds to investigate the specific action of lead upon the system. He considers, in the first place, the most important combinations which acetate of lead forms with the constituent parts of the animal economy; and, secondly, its relations to the animal solids and fluids. He next gives the results of experiments on animals in which the acetate was administered in large and small doses; in substance and in solution, or as forming a peculiar compound with albumen; and in which it was either introduced into the stomach or applied externally to wounds. The paper is concluded with the analysis of the blood and urine, and a general summary of the results of his experiments.

I. His first set of experiments, undertaken to ascertain the reciprocal decompositions of acetate of lead and the chief animal compounds, render it probable that the oxide of lead unites in different proportions with albumen and acetic acid to form definite compounds, some of which are soluble and others insoluble in water.

Several other organic principles enter into analogous combinations with lead. A few drops of acetate of lead produce in milk a copious precipitate of casein; and the filtered liquid, provided the milk was in excess, contains but a mere trace of lead. Salivin produces in solutions of acetate of lead a white precipitate, soluble in excess of salivin, completely soluble in muriatic acid, and which forms a troubled solution with acetic acid. Acetate of lead produces in solutions of osmazome a precipitate, insoluble in water and acetic acid, but soluble in excess of acetate of lead and in muriatic acid. With gelatine it forms soluble compounds, and with Schwann's digestive principle, prepared by digesting the mucous coat of the stomach of the calf in water acidified with muriatic acid, it forms a white precipitate insoluble in acetic, but soluble in muriatic acid.

From these statements it follows, that lead forms with casein a compound which in the stomach would be almost totally inert; while the compounds with salivin, osmazome, and the digestive principle would be called into activity by the action of the free acid of the gastric juice.

With mucus lead forms an insoluble compound, and it is probable most of the lead introduced into the stomach passes through the intestines in this inert combination. Lead does not appear to unite with fibrine, but it is rather probable that it forms with the red colouring matter of the blood a compound soluble in water.

When a muscle either dead or living is steeped in a solution of acetate of lead, it undergoes a chemical decomposition. The external layer becomes white, and gradually, but very slowly, the change extends to the deeper-seated fibres. A similar process takes place in the other organs of the body, as is daily seen in ulcers. A like decomposition ensues on every secreting surface between the secretion and the salt of lead, and only when the latter is in excess does it attack the subjacent organ. Consequently when small doses of acetate of lead are introduced into the stomach the salt is completely decomposed by the gastric secretions, and the mucous coat is not attacked; but when the doses are so large as only to be partially decomposed by the secretions of the stomach, the mucous coat of the stomach is attacked and corroded. A like corrosion ensues in all the organs of the body by similar doses; it is purely chemical, and takes place equally in the dead and living animal; but the decomposition is modified in the latter case in so far as life augments the secretion, and consequently favours the decomposition, and thus prevents in some measure the immediate contact of the metallic salt and secreting organ. Acetate of lead cannot, even for a moment, remain undecomposed in contact with the blood, or with any secretion, and cannot consequently exist as such in the circulation. When it is introduced into the stomach, decomposition, therefore, is an immediate result, and we have then no longer to do with the acetate, but with the newly formed compounds. Microscopical investigation is sufficient to show the decomposition of the serum of the blood by acetate of lead, but it is still problematical whether the globules suffer any change. A very small quantity of a metallic solution injected into a vein is sufficient to cause death, owing probably, judging from the appearances presented by the lungs, to the obstruction of their capillaries by the coagulation of the albumen of the serum.

II. In the experiments on animals, the solution of the acetate was introduced into the stomach by means of an elastic catheter. In the first class of experiments such quantities only of the salt were used as were sufficient to produce death without direct corrosion of the mucous membrane. To this end, in seven experiments upon rabbits of average size, half a gramme, or 8.21 grains, of the salt, dissolved in five parts of water, were injected into the stomach. From ten to twelve doses, (5 to 6 grammes, or 1 drachm 22 grains to 1 drachm 38.5 grains,) sufficed to produce death under remarkably similar symptoms and morbid appearances. The first doses produced little effect, the animals drank more than usual, took less food, and the fæces and urine were less in quantity. After the sixth or seventh dose there was grinding of the teeth; the animal lay frequently on the abdomen, but without the belly being painful on pressure. It now grew daily weaker, lay almost constantly on its belly, the inspiration was slow, and death followed in a paroxysm of opisthotonus. On opening the body, the stomach generally contained a quantity of an acid yellowish fluid, with a small quantity of white insoluble flakes. Lead was detected both in the fluid and flakes. The epithelium and mucus formed a thick, viscid, translucent homogeneous mass; the mucous coat itself was in general slightly attacked and marked with a large number of small white spots; but in one case the mucous coat of the stomach was unaltered, although the symptoms during life had been the same as in the other cases; thus proving that death was owing to the action of the newly formed compounds, and not to chemical corrosion. The small intestines, except in one case, were in their natural state; the large intestines were healthy and contained a small quantity of rather firm fæces, in which analysis shewed the presence of lead in such quantity as to prove that most of the lead had combined with the mucus to form an inert compound, which passes off with the excrements. The blood was evidently altered; it was of a peculiar cherry-red colour; the serum was proportionally small in quantity, and viscid; the coagulum was very firm. No lead could be detected in the blood by the above process of analysis.

The appearance in the lungs varied. In general they contained neither blood nor air, did not crepitate, but were often marked with dark patches, owing to small quantities of coagulated blood. In other cases they were very dark, contained black coagulated blood, but were not inflamed. The kidneys were healthy, the bladder generally filled with limpid urine. The other organs were natural. To study the effects of larger doses, ten grammes, or two drachms forty-four grains, of sugar of lead, dissolved in two parts water, were injected into the stomach. The respiration and circulation immediately became hurried, and continued so for some time. There was great thirst, and in general augmented excretions of fæces and urine. The urine was in general natural; often white, containing white insoluble flakes; sometimes bloody, but only during the first hours after the administration of the poison. In about an hour the respiration ceased to be frequent, and became rather slow and difficult; the animal was very weak, lay on the belly, and was often convulsed. It gradually became weaker, and died in a paroxysm of episthotonus, in a period varying from three to twelve hours.

In general the body was opened immediately after death. The mucous coat of the stomach was grey or white, dry and friable, and very like coagulated albumen or carein. It was easily separated, along with the layer of subjacent cellular tissue, from the muscular coat. The vessels of the cellular tissue were gorged with coagulated blood; the muscular coat was attacked in some places, but the peritoneum appeared healthy when the examination was made immediately after death. The whole tract of the small intestines shewed morbid appearances; the mucous coat was white, dry, and thickened: these alterations affected more particularly the villi, the intervening membrane being in general only spotted or very partially affected. The small intestines, besides, were often covered with red patches, which are not, however, to be ascribed to inflammation, but to the chemical action of the metallic salt. In such cases, from half an ounce to four ounces of bloody serum were found in the peritoneal cavity. The mucous coat of the large intestines was in no case affected, as the remainder of the undecomposed salt came in contact in the cæcum with a large quantity of fluid, by which it was perfectly decomposed, without affecting the subjacent tissues.

The changes in the blood were similar to those found in the cases in which small doses were administered, but the serum was in greater or less quantity according as the animal had drunk much or little. On opening the chest, the diaphragm was found high in that cavity, the lungs contracted and dark, with hardly any crepitation and almost entirely empty of air and fluid, containing only a small quantity of coagulated blood. When the urine was natural there was no change in the appearance of the kidneys; when it was troubled and milky their surface was in some places darker than natural, the cortical substance was in general very dark, and some of the cones of the tubular substance were also darker than natural as far as the papillæ. In cases where bloody urine was secreted the same changes were found, but in a more intense degree. The bloody urine is supposed to be dependent on decomposition of the blood.

When small doses of the salt of lead are exhibited death follows from the alteration of the blood; but the corrosion of the mucous membrane of the intestines is proved to have a powerful influence in accelerating the fatal termination. Five grammes, or one drachm twenty-two grains, of sugar of lead dissolved in two parts water, and injected into the stomach, were sufficient to cause death in some cases, but in others the effect was merely temporary. The symptoms were nearly the same as those already described, but bloody urine was a constant effect of these doses. When death was the consequence, the animal died in about forty-eight hours, and dissection showed extensive lesion of the mucous coat of the intestines, with alterations of the blood, lungs, and kidneys, similar to those found in the other cases. When the dose was not sufficient to cause death, the animal recovered its appetite, and passed limpid urine by the third day. It was then poisoned with prussic acid, and the lesions of the intestinal mucous coat were found on dissection to be unimportant. The rapidity with which death ensues is dependent, therefore, in a great measure upon the state of the mucous membrane, for the specific action of lead had taken place alike in both cases, as was sufficiently evident from the appearance of the urine.

It appears from the above experiments that acetate of lead produces corrosion of the mucous membrane only when the gastric fluids are not sufficient for its decomposition, and it is rendered probable that the peculiar poisonous action of lead results from the soluble compounds which it forms with the acids of the gastric juice. If this conjecture be correct, it is to be expected that the solution in acetic acid of the precipitate caused by albumen in a solution of the acetate will possess poisonous properties greater than those of the pure acetate. Experiments were made first with large doses: 2 grammes, or 32.8 grains, of the acetate were decomposed by albumen, and the precipitate, dissolved in acetic acid, was introduced into the stomach. Two such doses were sufficient to cause death, with symptoms in a great measure similar to those observed in the former cases. The mucous coat of the stomach was neither white nor corroded, but apparently quite healthy, except that in the large curvature there were some dark brown superficial spots, and that the mucous coat appeared thinner than natural. The villi were of a dark brown colour, but not inflamed. The alterations in the lungs, blood, and kidneys, were nearly the same as those found in the other experiments.

The experiment was then tried with small doses: half a gramme, or 8.21 grains, of the acetate were decomposed by albumen, and the resulting compound dissolved in acetic acid. This quantity was administered every alternate day. Seven doses produced death, and the animal died on the sixteenth day, having taken 57.37 grains of the salt of lead, only one-half of the quantity which is necessary to produce death when it is given in small doses as the pure acetate. The symptoms resembled very much those produced by small doses of the pure acetate. There was no corrosion found in the stomach; the mucous coat was thin, but firm, and had the appearance as if some of its component parts had been dissolved without change of form; the same dark coloured spots were observable in the large curvature as in the preceding case. The villi were somewhat reddened, and the colour depended upon injection of the veins. The lungs, kidneys, and blood presented the appearances already described.

It thus appears that the poisonous action of lead is produced more quickly and powerfully by this soluble compound than by the exhibition of the pure acetate, because in the latter case a large portion of the salt is rendered inert, by forming insoluble compounds with the contents of the stomach.

The above experiments were all performed upon rabbits, but the acetate was found to produce the same symptoms and lesions in dogs. Large doses of twelve grammes, or three drachms, seventeen grains, dissolved in three parts water, were introduced into the stomach of a dog, and the dose was repeated at various intervals, as its exhibition generally produced copious vomiting, by which most of the poison was ejected. On the fifth day the animal received the fourth dose, and appeared very weak and feeble on the sixth and seventh day. On the eighth day it received six grammes of the salt, after which it continued to become more and more feeble, and died on the eleventh day. The contents of the stomach were of a yellowish brown colour and were mixed with blood; the mucous coat was white and much corroded, and covered with cherry-red patches. The contents of the small intestines and cæcum resembled those of the stomach, but the mucous coat was no where corroded; it was partially reddened, owing to a gorged condition of the blood-vessels of the innermost layer of the mucous membrane. The lungs and blood were altered as in the previous experiments. The kidneys were healthy.

It is seldom that symptoms of poisoning follow from the external application of a metallic salt, because in external parts there exists no free acid to dissolve the compounds of the salt with the organic principles. Two grammes, or 32.8 grains, of acetate of lead, in substance, were introduced into the subcutaneous cellular tissue of a rabbit. Slight symptoms, resulting from mechanical irritation, followed. On the following day, two more grammes were introduced beneath another part of the skin, and two days later two more grammes in another part. No symptoms of poisoning followed, the animal continued to take its food, and the excretions were natural. The symptoms of mechanical irritation, however, were now considerable, and the animal died in consequence on the evening of the fifth day. The cellular tissue, muscles, tendons, sheaths, membranes, &c. were found changed into a dry white friable mass, extending to a depth of from one to three lines. The whole of the abdominal viscera were healthy. The blood was natural, and the lungs, although firmer than usual, were not so to such a degree as to warrant the conclusion of an affection of the system by lead.

A solution of half a gramme (8.21 grains,) of the acetate was decomposed by albumen, and the precipitate, dissolved in a slight excess of acetic acid, was injected into the cellular tissue beneath the skin of another rabbit. The animal very soon became dull, the respiration hurried, and belly contracted. The thirst was great, the animal lay upon its belly, and died in a paroxysm of opisthotonus in about twenty hours. On dissection most of the solution was found in the cellular tissue. The neighbouring parts were altered and discoloured, were softer, drier, and more friable than natural. The intestinal canal was injected, but otherwise healthy. The blood had undergone the same changes as in the other cases of poisoning by lead, the kidneys were injected, the lungs darker than usual, and contained a very small quantity of blood and air. No other material change was observable. In another case one gramme of the acetate applied in a similar manner produced death in three hours.

III. Dr. Mitscherlich was unable by the most minute analysis to prove satisfactorily the presence of lead in the blood or urine of animals poisoned by lead, and he therefore infers that if it passes at all into the blood or urine it must be in very small quantities only.

In his closing remarks he gives a summary of his results, and points out the practical utility of his experiments, and concludes by promising a series of articles upon the action of other metallic salts upon the system, to be followed by others

P 2

upon the action of the alkalies, earths, alcaloids, and other organic principles.—*Müller's Archiv. für Anatomie und Physiologie. Jahrgang,* 1836. Heft iv. and v.

---

*On the Origin of the Fifth and Seventh Pair of Nerves.* By A. Retzius.

The olivary bodies seem to stand in close relation with the facial nerves, 1stly, because these nerves arise from the medulla oblongata immediately anterior to the olivary bodies; in the brain of the fœtus, which consists almost entirely of a reddish gray mass, containing some narrow white bands in the situation of the principal cords, there is one such band which passes from the seventh pair to the upper edge of the olivary body, or the funicular siliquæ of Burdach; 2dly, because the roots of the seventh pair can be traced in adults likewise to the external fibrous bundles, and upper edge of the olivary bodies; 3dly, because the development of the seventh pair of nerves seems to be dependent upon that of the olivary bodies, as both are found most perfect in man, and are only imperfectly developed in the lower animals.

It is proved by comparative, experimental, and pathological anatomy, that the functions of the seventh pair are to produce the expressive (*mimische*) motions of the countenance; and as these, together with the motions which are produced in speaking, (both being peculiar attributes of the human race,) are excited, by means of the facial and hypoglossal nerves, from the olivary bodies, we are warranted in assuming these bodies as the central organ of the motions of expression (*mimische Bewegungen,*) and speech.

From late researches it appears that the motor branch of the fifth pair of nerves really arises from the pyramidal bodies, from the fibrous bundles, namely, which lie in the pons varolii; and that the branch for sensation arises from the interior of the corpora restiformia. As the pyramids belong to the motor, and the restiform bodies to the sensory apparatus, it follows that this double origin of the nerve corresponds with the double origin of the spinal nerves, and harmonizes with the fundamental idea of Bell's system.—*Müller's Archiv. für Anatomie und Physiologie. Jahrgang,* 1836. Heft v.

